# *In vitro *and *ex vivo *effect of hyaluronic acid on erythrocyte flow properties

**DOI:** 10.1186/1423-0127-17-8

**Published:** 2010-02-12

**Authors:** A Luquita, L Urli, MJ Svetaz, AM Gennaro, ME Giorgetti, G Pistone, R Volpintesta, S Palatnik, M Rasia

**Affiliations:** 1 Cátedra de Física Biológica, Facultad de Ciencias Médicas, Universidad Nacional de Rosario, Santa Fe 3100, 2000 Rosario, Argentina; 2 Sección Inmunidad Celular, Department of Bioquímica Clínica, Universidad Nacional de Rosario, Facultad de Cs. Bioquímicas y Farmacéuticas, Suipacha 531, 2000 Rosario, Argentina; 3 Facultad de Bioquímica y Ciencias Biológicas, Universidad Nacional del Litoral (UNL) and INTEC (CONICET-UNL), Güemes 3450, 3000 Santa Fe, Argentina; 4 Área Reumatología, Cátedra de Reumatología, Facultad de Ciencias Médicas, Universidad Nacional de Rosario, Santa Fe 3100, 2000 Rosario, Argentina

## Abstract

**Background:**

Hyaluronic acid (HA) is present in many tissues; its presence in serum may be related to certain inflammatory conditions, tissue damage, sepsis, liver malfunction and some malignancies. In the present work, our goal was to investigate the significance of hyaluronic acid effect on erythrocyte flow properties. Therefore we performed *in vitro *experiments incubating red blood cells (RBCs) with several HA concentrations. Afterwards, in order to corroborate the pathophysiological significance of the results obtained, we replicated the *in vitro *experiment with *ex vivo *RBCs from diagnosed rheumatoid arthritis (RA) patients, a serum HA-increasing pathology.

**Methods:**

Erythrocyte deformability (by filtration through nucleopore membranes) and erythrocyte aggregability (EA) were tested on blood from healthy donors additioned with purified HA. EA was measured by transmitted light and analyzed with a mathematical model yielding two parameters, the aggregation rate and the size of the aggregates. Conformational changes of cytoskeleton proteins were estimated by electron paramagnetic resonance spectroscopy (EPR).

**Results:**

*In vitro*, erythrocytes treated with HA showed increased rigidity index (RI) and reduced aggregability, situation strongly related to the rigidization of the membrane cytoskeleton triggered by HA, as shown by EPR results. Also, a significant correlation (r: 0.77, p < 0.00001) was found between RI and serum HA in RA patients.

**Conclusions:**

Our results lead us to postulate the hypothesis that HA interacts with the erythrocyte surface leading to modifications in erythrocyte rheological and flow properties, both *ex vivo *and *in vitro*.

## Background

Elevated seric hyaluronic acid (HA) is a feature of certain inflammatory conditions, notably rheumatoid arthritis and scleroderma, and also accompanies tissue damage, sepsis, liver malfunction and some malignancies [1-8].

Additionally, the employment of HA is currently suggested in the therapy of arthritis, arthrosis, psoriasis, and it is also included in treatments with cosmetic products [9-12].

Being HA a macromolecule present in plasma, it could interact with the red blood cell (RBC) surface, as it happens with albumin. In a previous work [13] we have demonstrated that albumin adsorption impairs erythrocyte rheology in a concentration-dependent fashion increasing the erythrocyte rigidity index (RI). Such facts lead us to hypothesize that the reduction in erythrocyte deformability (RI increase) observed in serum HA-increasing pathologies, could be due to HA interaction with RBC surface which contributes to the impaired flow properties observed in these pathologies [14,15].

We therefore conducted this study to investigate the significance of serum HA effect on erythrocyte flow properties.

We performed *in vitro *experiments incubating RBCs from healthy donors with several HA concentrations. Afterwards, in order to corroborate the obtained results, we selected a serum HA-increasing pathology and replicated the experiment *ex vivo *with RBCs from those patients. We chose rheumatoid arthritis RA patients because in an earlier paper we demonstrated a reduction in erythrocyte RI that is in close correlation with the Disease Activity Score (DAS 28-4) index during the clinical remission of the process [16].

## Methods

The Ethics Committee of the Facultad de Ciencias Médicas, Universidad Nacional de Rosario, Argentina approved the study protocol, and all participants signed an informed consent according to the recommendations of the Declaration of Helsinki [17].

### Blood sample collection and laboratory assays

Blood samples of RA patients were obtained by venipuncture and separated in 2 aliquots. One of them was collected in tubes containing EDTA and assigned to determine haematimetric indexes, plasmatic protein concentration and rheological parameters. The other was collected in a dry tube and centrifuged 5 min at 5000 RPM in order to obtain serum for the serum concentration of HA.

a) *Haematimetric indexes*: Erythrocyte count was assessed by a hæmocytometer and hæmoglobin by the cyanmetahæmoglobin method. From these values, MCV and MCHC were calculated.

b) *Plasmatic immunoglobulin concentration*: by radial immunodiffusion.

c) *Fibrinogen concentration*: by commercial kinetic test kit (Boehringer Mannheim, Germany).

d) *HA *assay: by an ELISA commercial test kit (CHUGAI quantitative test Kit), using HABP (HA Binding Protein) as capture molecule [18].

### Haemorheological assays

#### a) Rigidity index (RI)

Whole blood from RA patients was centrifuged at 5000 RPM for 5 minutes, plasma and buffy coat were separated and the erythrocytes were washed twice with PBS (0.12 M NaCl, 0.03 M H_2_KPO_4_/HNa_2_PO_4 _with 2 mg/ml glucose).

Washed RBCs were resuspended (10% hæmatocrit) in PBS with bovine albumin (0.25%) (Sigma Chemical Co., St.Louis, MO, USA) in order to prevent erythrocyte aggregation.

Erythrocyte filtration was performed in a computerized instrument using the Reid et al. technique [19]. Briefly, a 10% suspension of washed erythrocytes was passed through a polycarbonate filter, 5 μm pore size (Nucleopore Corp. USA), using a negative filtration pressure of 10 cm H_2_O. The flow time required for 1 ml of RBC suspension to pass through the filter was measured. Results were expressed as the rigidity index (RI) that is an estimation of erythrocyte rigidity (inverse of erythrocyte deformability) [20], defined as:

Where: Tb: time of passage of the cell suspension through the filter; Ts: time of passage of an equal volume of PBS; Htc: hæmatocrit (10%).

The erythrocyte deformability measurements are in accordance to the International Committee for Standardization in Haematology [21].

#### b) Erythrocyte aggregation

This parameter was measured in whole blood at native hæmatocrit. An instrument [22] assembled as a model designed by Tomita et al. [23] was used. In brief, it consists of a densitometer head that detects light transmission changes in whole blood during the aggregation process that follows a disaggregating agitation [24].

The registered data were analyzed with a mathematical model allowing us to determine two parameters: 2k_2_n_0_, which stands for the initial rate of the process, and s_0_/n_0_, which estimates aggregation intensity and average rouleaux size at process completion.

#### c) Erythrocyte membrane fluidity

Erythrocyte membrane fluidity was estimated by electron paramagnetic resonance spectroscopy (EPR) using liposoluble spin labels 5, 12 and 16- doxyl stearic acid (5-, 12-, and 16-SASL, Sigma Chemical Co., St. Louis, MO, USA), which sense the mobility of the acyl chains at different depths in the lipid leaflet of the RBC membrane [25]. The EPR spectra were obtained at 25 ± 1°C in a Bruker ER-200 spectrometer operating at X band (9800 MHz).

In the case of erythrocytes from RA patients, membrane fluidity was assessed using the parallel component of the nitrogen hyperfine tensor of 5-SASL (T_//_) as a representative parameter of lipid chain rigidity. Thus, increased T_// _values are indicative of decreased membrane fluidity [26].

In the case of cells incubated in vitro with HA, we evaluated S_5_, S_12 _and S_16 _order parameters at different depths of the lipid bilayer, from the spectra of 5, 12 or 16-SASL. As in the previous case, increased S parameters indicate decreased membrane fluidity.

### HA purification

HA was purified from other acid mucopolysaccharides by ecteola cellulose chromatography [27] and eluted with 0.05 N HCl. HA concentration in the eluate was colorimetrically determined, through estimation of the glucuronic acid content, by using carbazole in sulphuric medium [28].

The elution solution was neutralized to pH 7.4 with 0.05 N NaOH

### *In vitro *experiments

#### - Erythrocyte incubation in hyaluronic acid solutions and RI determination

Blood samples were obtained from healthy adults by venipuncture and collected in tubes containing EDTA (1,146 mg/ml, Sigma Chemical Co., St.Louis, MO, USA) as anticoagulant. Each sample was fractioned in 5 aliquots (1 ml). The first sample (control; n = 6) was additioned with 1 ml of neutralized elution solution and the other ones with 1 ml of purified HA in rising concentrations, yielding the following final nominal concentrations (μg/ml): [HA_1_] = 50; [HA_2_] = 87; [HA_3_] = 109; [HA_4_] = 190 (n = 6). After 30 min incubation at 37°C, serum HA concentration ([HA]s) and erythrocyte RI were determined for each sample in a similar way as for RA patients.

#### - Reversibility of HA-erythrocyte interaction

In order to test the reversibility of HA-erythrocyte interaction, RI was determined again in erythrocytes of each sample after washing twice with PBS.

#### -Aggregability determination in erythrocytes incubated in HA

Blood samples were divided into two fractions; one of them was added with purified HA to reach a final concentration similar to that found in serum of RA patients, [HA] = 109 μg/ml (HA group; n = 15), and the other one was added with the same volume of the the neutralized elution solution (control group; n = 15). Both aliquots were incubated for 30 min at 37°C. Afterwards, serum concentration of HA was determined and erythrocyte aggregability was measured as described previously.

##### EPR spin label studies of the cytoskeleton proteins in haemoglobin-free erythrocyte membranes

In order to obtain haemoglobin-free erythrocyte membranes, RBC's from regular donors were subjected to hypotonic lysis in sodium phosphate buffer 5 mM, pH 8 (for 30 min at 4°C) and the pellet was thoroughly washed [29]. The membrane samples were subdivided into two fractions. One of them (HA group; n = 6) was added with purified HA to reach concentrations similar to those found in serum of RA patients, and the same volume of the elution solution was added to the other fraction (control group; n = 6). Both media had been previously neutralized to pH 7.4.

Both aliquots were incubated with the spin label 4-maleimido-Tempo (Mal-Tempo, Sigma Chemical Co., St.Louis, MO, USA), at a concentration of 30-50 μg per mg of protein, in the dark, at 4°C for 1 h.

The protein-specific spin-label Mal-Tempo is known to bind covalently to cysteine sulfhydryl groups of cytoskeleton membrane proteins. W/S parameter, estimated from the Mal-Tempo EPR spectrum [29], reflects two types of membrane protein SH-binding sites for the spin label: strongly and weakly immobilized sites (S and W sites, respectively). Changes in the W/S parameter are indicative of conformational changes in the cytoskeleton proteins.

### *Ex vivo *experiments

#### -RA Patients

One hundred female RA patients attending an outpatient service at the Departamento de Reumatologia, Universidad Nacional de Rosario, Argentina, were included in the present study (mean age 48 ± 17 yr).

The patients were part of a follow-up study recruited between the years 2000 and 2003 [13]. RA diagnosis was established following the American College of Rheumatology criteria [30-32]. Patients with cardiovascular or liver disease, cancer, chronic infectious diseases, HIV positive serology or diabetes mellitus as well as heavy smokers (>20 cigarettes/day) and patients who were under medication that could alter hæmorheological blood properties, were dismissed. The laboratory process has been described previously [13]. The clinic activity of the disease was evaluated by means of the Disease Activity Score (DAS 28-4) [33].

#### Controls

The control group consisted of 40 female non-smoker healthy volunteers, age-matched (mean: 43 ± 12 yr).

### Statistical analysis

**Figure 1:**
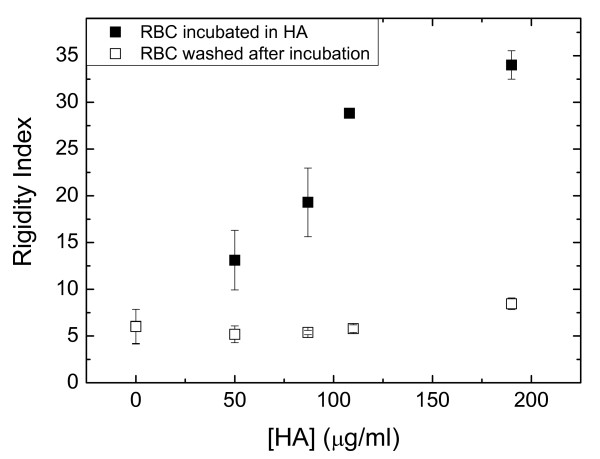
**Rigidity Index (RI) of erythrocytes incubated *in vitro *with variable HA concentrations, and reversibility of HA effect**. *In vitro *effect of several.hyaluronic acid (HA) concentrations on rigidity index (RI). Each sample was fractioned in 5 aliquots (1 ml). The first sample (control; n = 6) was additioned with 1 ml of neutralized elution solution and the other ones with 1 ml of purified HA in raising concentrations, yielding the following final nominal concentrations (μg/ml): [HA_1_] = 50; [HA_2_] = 87; [HA_3_] = 109; [HA_4_] = 190 (n = 6). As can be seen, after two washings, RI returns to control values. Data: median, C.I._95%_: confidence interval. (n = 6). RI: Kruskal Wallis' test: H = 27.87; p < 0.0001. *Post hoc *tests were performed with Mann-Whitney's U between groups, p < 0.05. RI after wash: Kruskal Wallis' test n.s.

The Kruskal-Wallis' test was performed considering variables; RI: RI after washes; afterwards Mann-Whitney's U test was applied as *post hoc *one. Wilcoxon's test was performed between RI and RI after washes for each group. Data are presented as median and 95% confidence interval (Figure 1).

Comparisons for aggregation parameters (2k_2_n_0 _and s_0_n_0_) between HA and control groups were performed by Student's t-test for paired data. Values are presented as mean ± standard deviation (Table 1).

**Table 1:** Erythrocyte aggregability in the presence of HA and its control (n = 15)

	2k_2_n_0_	s_0_/n_0_
Control	1.98 ± 0.14	1.867 ± 0.015
	
HA Group	1.29** ± 0.21	1.866 ± 0.004

Data: mean ± standard deviation.

Degree of significance of the difference between groups: ** p < 0.01

Differences in cytoskeleton protein conformation and in lipid chain ordering at different levels of the membrane between control and HA treated erythrocytes, assesed by EPR, were analized using Wilcoxon test for paired data. Results are expressed as median and 95% confidence interval (Table 2).

**Table 2:** HA effect on cytoskeleton protein conformation and on lipid chain order at different levels

	[HA] μg/ml	W/S	S_5_	S_12_	S_16_
Control	< 10	3.20 (3.10 -- 3.30)	0.693 (0.685--0.703)	0.525 (0.524--0.527)	0.230 (0.228--0.230)

HA Group	103 (100-105)	2.65* (2.60 --2.70)	0.690 (0.677--0.707)	0.521 (0.520--0.524)	0.229 (0.225--0.233)

W/S: ratio of the spectral amplitudes of Mal-Tempo attached to strongly and weakly immobilized sulfhydryl groups.

S_5_, S_12 _and S_16_: 5, 12 or 16- doxyl stearic acid spin labels.

Data: median, C.I._95%_: confidence interval. (n = 6).

Degree of significance of the difference between groups: * p < 0.05

The correlation between RI and [HA]s in RA patients was estimated using Pearson product-moment correlation coefficient. Values were presented as mean ± standard deviation (Table 3).

**Table 3:** Rigidity index and hyaluronic acid concentration in patients with active Rheumatoid Arthritis and their controls

	[HA]s (μg/ml)	RI
Controls (n = 40)	20.0 ± 9.0	7.0 ± 0.8

RA Patients (n = 100)	155.80 ± 44.0	11.0 ± 1.3

P	< 0.00001	< 0.001

[HA]s: serum concentration of hyaluronic acid; RI: rigidity index; RA: rheumatoid arthritis.

Pearson product-moment correlation coefficient was also used to analyze the dependence of RI with [IgG], [IgM], MHCM, T_// _and fibrinogen concentration in RA patients.

## Results

### *In vitro *experiments

Figure 1 shows that the rigidity index is significantly increased after incubation with HA at all the measured [HA] range. There is a remarkable good correlation between RI and [HA]s (r_s_: 0.996, p < 0.00001). Figure 1 also shows that after two washings, RI returns to control values. Thus, it can be postulated that HA reduces erythrocyte deformability reversibly and in a concentration dependent manner.

Regarding aggregation properties, the results presented in Table 1 indicate a significant decrease in the parameter 2k_2_n_0 _in erythrocytes incubated with HA, while no differences in the parameter s_0_/n_0 _are observed. This means that the presence of HA in the incubation medium diminishes the erythrocyte aggregation rate, without significantly modifying the size of the aggregates.

Table 2 shows that the order parameters S, calculated from the EPR spectra of liposoluble spin labels, do not exhibit significant differences between HA group and control group, indicating that the fluidity of the lipid bilayer is not altered as a consequence of the presence of HA. Conversely, the W/S parameter, calculated from the spectra of Mal-Tempo, was significantly diminished in the HA group. This suggests that incubation with HA introduces changes in the conformation of the cytoskeletal protein spectrin.

### *Ex vivo *experiments

Previous analysis [16] performed on erythrocytes from active RA patients (DAS 28-4 > 2,6) showed a good correlation between disease activity and serum HA concentration [HA]s (Pearson product-moment correlation coefficient (r) DAS 28-4 vs. [HA]s: 0.87, p < 0.0001). Table 3 shows that erythrocytes from the active RA patients have a rigidity index significantly higher than those of the control group, together with a significantly increased [HA]s.

Subsequent correlation analyses were performed between erythrocyte RI and intrinsic and extrinsic parameters. It was found that RI has a significant correlation with [HA]s (r: 0.77 p < 0.00001), while it does not correlate either with lipid bilayer rigidity (T_//_) or with internal viscosity (evaluated through MCHC). Also, there was no significant correlation between RI and plasma proteins, namely, IgG (r: 0.32, p > 0.05) and IgM (r: 0.33, p > 0.05), and fibrinogen (r: 0.12, p > 0.05), which might be adsorbed on cell surface modifying the membrane rheology.

## Discussion

Erythrocyte rigidity is a determining factor concerning flow resistance, especially in microcirculation, where RBCs must pass through capillaries of a diameter lower than the cells. Even in macrocirculation, rigidity is a factor of flow resistance, thus contributing to the hiperviscosity syndrome.

HA is a glycosaminoglycan --a high molecular weight polysaccharide--that, similarly to albumin, could interact with the erythrocyte surface. Our hypothesis was that this interaction could lead, in the same way that albumin does, to a reduction in the flexibility of the membrane. The verification of this hypothesis demanded to establish a correlation between RI values and HA medium concentration.

When blood from healthy donors was incubated with several HA concentrations it was corroborated that HA caused a significant decrease in erythrocyte deformability (increase in RI) in a concentration-dependent manner and reversibly-- this effect was reverted by washing the treated cells.

In an earlier paper [16] we have demonstrated that RBC's from RA patients presented a considerably increased RI. In the same paper [16] it was corroborated that RI is a reliable indicator for RA activity, given its significant correlation with DAS 28-4 score.

Experiments performed on blood from RA patients in different levels of activity of the disease showed that HA was the only plasma factor that significantly affected deformability; moreover, the expected correlation between RI values and [HA]s was found (r: 0.77, p < 0.00001). The discrepancy of RI values in erythrocytes of RA patients (Table 3) with those of erythrocytes incubated with similar HA levels in the in vitro experiment (Figure 1) should be attributed to the presence in plasma of pathology dependent factors affecting the erythrocyte rheology.

One of the techniques classically employed in RA diagnosis is erythrocyte sedimentation rate (ESR). This value estimates mainly the rise in erythrocyte aggregation. Rouleaux formation depends on medium and cell factors. Consequently, its increase may be explained by the rise in fibrinogen and/or globulin concentration and/or to the modification of the erythrocyte surface properties.

In our *in vitro *experiments, it was observed that HA-treated RBCs showed a lower aggregation rate (Table 1), i.e., a lower tendency to form rouleaux in comparison to controls. This fact implies that the increased ESR in blood from RA patients could only be attributed to modifications in plasma proteins and not to cell factors [33].

It has been proved that albumin --the smallest and most important plasma protein-- is adsorbed on the erythrocyte surface [13] and, unlike globulins and fibrinogen, hinders the aggregation process. The corroboration that HA presented a similar behaviour to that of albumin constitutes a further support for the claimed hypothesis.

EPR spectroscopy allowed us to investigate the effects caused by the *in vitro *interaction of HA with RBC membrane. As shown in Table 2, order parameters did not change significantly, indicating that the fluidity of the lipid bilayer was not altered as a consequence of HA incubation. On the contrary, the parameter W/S, calculated from the spectrum of a protein spin label, revealed that HA produces alterations in the spectrin structure of the membrane cytoskeleton increasing the amount of strongly immobilized sites. This result suggests that the increase in erythrocyte rigidity is related to a stiffening of the cytoskeleton. However, as HA only interacts with the outer erythrocyte surface, we postulate that HA interaction results in a protein organizational perturbation that is translated to spectrin in the inner membrane surface.

## Conclusions

Our experiments lead us to accept the hypothesis that HA interacts with the erythrocyte surface, giving place to modifications in erythrocyte rheological and flow properties.

Considering that HA is increased in inflammatory processes and other malignancies, and that it is employed in pharmacologic and cosmetic treatments, in all these cases we claim that the effect of HA upon erythrocytes --and thus on circulatory function-- should not be disregarded; in fact, special attention should be paid to this matter.

## Abbreviations

RA: rheumatoid arthritis; HA: hyaluronic acid; RI: rigidity index; DAS: Disease Activity Score; HIV: human immunodeficiency virus; EDTA: ethylenediaminetetraacetic acid; ESR: erythrocyte sedimentation rate; RBC: red blood cell; PBS: phosphate buffered saline; MCV: mean corpuscular volume; MCHC: mean corpuscular hæmoglobin concentration; EPR: electron paramagnetic resonance spectroscopy; SH: sulfhydryl groups; [HA]s: serum concentration of hyaluronic acid; T_//_: nitrogen hyperfine tensor; S_5_, S_12 _and S_16_: EPR order parameters determined using 5, 12 or 16- doxyl stearic acid spin labels.

## Competing interests

The authors declare that they have no competing interests.

## Authors' contributions

AL: acquisition, analysis and interpretation of data of haemorheological assays in RA patients and HA purification by ecteola cellulose chromatography. Also involved in drafting the manuscript, revising it critically and in giving final approval of the version to be published. LU: acquisition, analysis and interpretation of data of laboratory assays in RA patients. MJS: Blood sample collection in RA patients and HA assay by an ELISA commercial test kit. AMG: acquisition, analysis and interpretation of data of erythrocyte membrane fluidity estimated by electron paramagnetic resonance spectroscopy (EPR). MEG: acquisition, analysis and interpretation of data of erythrocyte aggregation of the in vitro experiments. GP: acquisition, analysis and interpretation of data of erythrocyte deformability of the in vitro experiments. RV and SP: protocol design and obtention of the consent de RA patients. Determiantion of the clinic activity of the disease, evaluated by means of the Disease Activity Score (DAS 28-4). MR: involved in drafting the manuscript and revising it critically, and giving final approval of the version to be published. All authors read and approved the final manuscript.
